# Total RNA extraction from tissues for microRNA and target gene expression analysis: not all kits are created equal

**DOI:** 10.1186/s12896-018-0421-6

**Published:** 2018-03-16

**Authors:** Rikki A. M. Brown, Michael R. Epis, Jessica L. Horsham, Tasnuva D. Kabir, Kirsty L. Richardson, Peter J. Leedman

**Affiliations:** 1 Laboratory for Cancer Medicine, Harry Perkins Institute of Medical Research, University of Western Australia, Centre for Medical Research, QEII Medical Centre, 6 Verdun St, Nedlands, WA 6009 Australia; 2 School of Medicine and Pharmacology, the University of Western Australia, Nedlands, WA 6009 Australia

**Keywords:** microRNAs, microRNA isolation method, Biomarkers, miRNA-based therapy, Extraction, Real-time PCR, Tissue

## Abstract

**Background:**

microRNAs (miRNAs) are short non-coding RNAs that fine-tune gene expression. The aberrant expression of miRNAs is associated with many diseases and they have both therapeutic and biomarker potential. However, our understanding of their usefulness is dependent on the tools we have to study them. Previous studies have identified the need to optimise and standardise RNA extraction methods in order to avoid biased results. Herein, we extracted RNA from murine lung, liver and brain tissues using five commercially available total RNA extraction methods. These included either: phenol: chloroform extraction followed by alcohol precipitation (TRIzol), phenol:chloroform followed by solid-phase extraction (column-based; miRVana and miRNeasy) and solid-phase separation with/without affinity resin (Norgen total and Isolate II). We then evaluated each extraction method for the quality and quantity of RNA recovered, and the expression of miRNAs and target genes.

**Results:**

We identified differences between each of the RNA extraction methods in the quantity and quality of RNA samples, and in the analysis of miRNA and target gene expression. For the purposes of consistency in quantity, quality and high recovery of miRNAs from tissues, we identified that Phenol:chloroform phase separation combined with silica column-based solid extraction method was preferable (miRVana microRNA isolation). We also identified a method that is not appropriate for miRNA analysis from tissue samples (Bioline Isolate II). For target gene expression any of the kits could be used to analyse mRNA, but if interested in analysing mRNA and miRNA from the same RNA samples some methods should be avoided.

**Conclusions:**

Different methods used to isolate miRNAs will yield different results and therefore a robust RNA isolation method is required for reproducibility. Researchers should optimise these methods for their specific application and keep in mind that “total RNA” extraction methods do not isolate all types of RNA equally.

**Electronic supplementary material:**

The online version of this article (10.1186/s12896-018-0421-6) contains supplementary material, which is available to authorized users.

## Background

microRNAs (miRNAs) are a family of short (~ 22 nucleotide) non-coding RNAs that are essential for controlling the regulation of gene expression. They act via binding to specific sequences in the 3′-untranslated region (3′-UTR) of mRNAs, leading to either translational repression or message decay. miRNAs have been linked to distinct biological processes in normal development and their altered expression is implicated in many diseases including neurological disorders, immune diseases and cancers [1]. miRNA profiling studies have helped to elucidate their critical roles in developmental biology such as regulation of cell cycle [2], apoptosis [3] and differentiation [4]. Furthermore, their altered expression in disease makes them attractive as diagnostic and prognostic biomarkers, and as targets for manipulation as therapeutics.

With the increasing interest in miRNAs, particularly as diagnostic and prognostic biomarkers, a number of platforms have been developed to analyse miRNA expression. Current detection methods of miRNAs involve cloning, in situ hybridisation, microarray, bead-based flow cytometry, northern blot, next generation sequencing and quantitative real time PCR (qRT-PCR) [5, 6]. These methods each have their advantages and disadvantages. However, all rely on the quality of the in-put material. Detection methods such as qRT-PCR and microarray are dependent on the assumption that the RNA extraction method isolates all miRNAs equally [7–9]. It is becoming evident that here is a crucial need to optimise and standardise the isolation of miRNA, given the discrepancies among many studies, whereby the recovery of miRNAs is dependent on the extraction method used [10–16]. These initial studies have focussed on miRNA recovery from plasma, urine and other bodily fluids, however to date few papers have compared differences in miRNA extraction efficiency from solid tissues.

The development of a miRNA for therapeutic use also requires knowledge of its specific gene targets, a field of research which is in its infancy. Therefore, it may be of specific importance for researchers to analyse both miRNA and target gene expression in the same RNA sample. However, different extraction methods may bias towards isolation of either long or short RNAs, potentially impacting the findings of the study. This research used five commonly used RNA isolation methods to extract total RNA including miRNAs from solid tissues, and compared the extraction efficiency, quality and yield of resultant RNAs. We analysed miRNA and target gene expression using qRT-PCR from brain, liver and lung tissues from mice. The data presented herein could serve as a useful reference for researchers interested in analysing both miRNA and target gene expression in animal tissues for diagnostic and prognostic use or miRNA validation.

## Methods

**Fig. 1:**
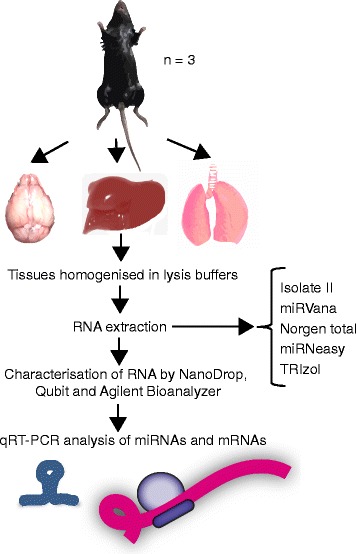
Experimental design for comparing RNA isolation methods from mouse tissues. Brain, lung and liver tissues were dissected from C57BL/6 mice (*n* = 3) and rapidly frozen. Tissues were homogenised and RNA extracted using five different methods and assessed for yield, purity, integrity and miRNA abundance. miRNA and target gene expression was then quantitated with qRT-PCR

The overall workflow for the study is outlined in Fig. 1.

### Biological materials

Brain, liver and lung tissue samples were from 8 to 10 week old male C57Bl/6 mice (*n* = 3). Mice were euthanised by cervical dislocation, then tissue samples were harvested and immediately snap frozen using 100% ethanol and dry ice, and stored at − 80 °C until used. The use of mice was in accordance with local institutional ethical guidelines.

### RNA isolation

**Fig. 2:**
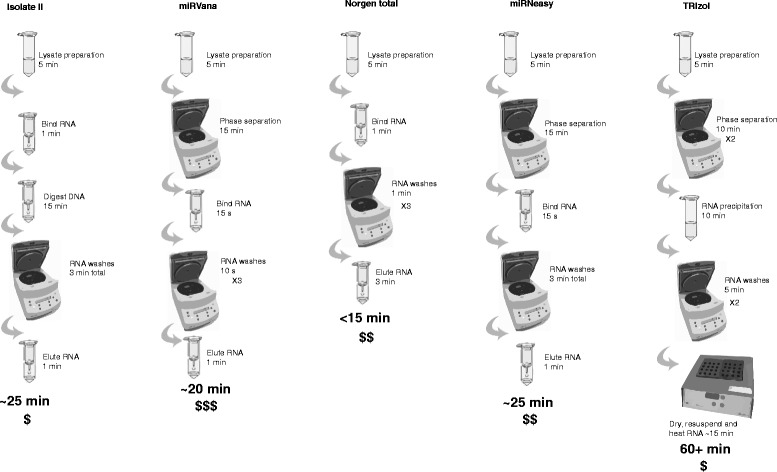
Flow chart summarising differences in the protocols, time requirements and prices of the different RNA purification methods. RNA was isolated using five methods that were either phenol based (TRIzol), column-based (Isolate II and Norgen total), or combined phenol and column-based methods (miRNeasy and miRVana). $ < 5, $$ = 5–10, $$$ > 10 AUD$ per sample preparation. Time requirement based on following instructions provided by manufacturer (not taking into account extra time required by the technician to perform the tasks)

Total RNA was isolated using the following kits: Isolate II RNA mini kit (Bioline, London, UK), miRVana microRNA isolation kit (Thermo Fisher Scientific, Waltham, MA, USA), Total RNA isolation kit (Norgen Biotek, Thorold, ON, Canada), miRNeasy mini kit (Qiagen, Hilden, Germany), and TRIzol Reagent (Thermo Fisher Scientific). Frozen tissue samples were weighed and processed on ice to prevent thawing. In preparation for RNA extraction 5–10 mg of tissue (weights differed by tissue but were consistent for all kits) was added to 700 μl of the appropriate lysis buffer. Samples were homogenised using a TissueLyser II (Qiagen) with 5 mm stainless steel beads for 3 × 1 min cycles at 30 Hz, with resting on ice in between. Homogenates were subjected to a single freeze/thaw cycle to aid cell lysis and then cleared by centrifugation at 10,000 x g for 5 min at 4 °C. RNA extractions were performed according to manufacturer’s instructions, with the exception of an additional chloroform extraction step for the TRIzol reagent protocol as illustrated in Fig. 2. RNA was eluted in 35 μl of RNase free water and stored at − 80 °C. Note that for all methods the total RNA extraction method was followed. miRNA enrichment was not performed for any method as efficient isolation of both miRNA and mRNA transcripts from the same sample is of interest.

### RNA quantity and quality assessment

RNA samples were quantitated using the Qubit HS RNA kit (Thermo Fisher Scientific) with a Qubit 3.0 Fluorometer (Thermo Fisher Scientific), following manufacturer’s instructions. The 260/280 and 260/230 ratios of absorbance values were used to assess the purity of RNA using a Nanodrop ND-1000 spectrophotometer (Thermo Fisher Scientific). A 260/280 ratio of ~ 2.0 and 260/230 ratio in the range of 2.0–2.2 was accepted as “pure” for RNA. Lower ratios may indicate the presence of protein, phenol, EDTA, carbohydrates or other contaminants that absorb at or near 260, 230 or 280 nm. The miRNA content was quantitated using a Qubit microRNA assay kit (Thermo Fisher Scientific) with a Qubit 3.0 Fluorometer, according to manufacturer’s recommendations. The integrity of the RNA was determined for all samples using an Agilent Bioanalyzer 2100 (Agilent Technologies, Diegem, Belgium) with an Agilent RNA 6000 kit (Agilent Technologies). RNA Integrity Numbers (RINs) were used to evaluate the integrity of the RNA samples with > 7.0 considered intact and < 7.0 considered degraded. The quality assessment of RNA samples is provided in Additional file 1: Figure S2, Additional file 2: Figure S3, Additional file 3: Figure S4.

### Reverse transcription and quantitative real time PCR (qRT-PCR)

For miRNA detection, 10 ng of the RNA samples were reverse transcribed in technical duplicates using a TaqMan microRNA reverse transcription kit (Thermo Fisher Scientific) with Taqman microRNA primers specific for the miRNAs of interest, following manufacturer’s instructions (see Additional file 4: Table S1). Reverse transcription products were diluted to 50 μl and 5 μl of diluted sample used in single qPCR reactions, with a total volume of 20 μl. qRT-PCR was performed using Taqman microRNA assays (Thermo Fisher Scientific) and TaqMan universal PCR master mix II, no UNG (Thermo Fisher Scientific) on a ViiA7 Real-Time PCR system (Applied Biosystems/Thermo Fisher Scientific), using recommended PCR cycling conditions. For analysis of target gene mRNA expression 250 ng of RNA was reverse transcribed into cDNA using a Quantitect reverse transcription kit (Qiagen) in duplicate reactions. For qRT-PCR 2 μl of cDNA was added to SensiMix SYBR Hi-ROX (Bioline), Quantitect Primer Assays (Qiagen; see Additional file 4: Table S1) and made up to a final volume of 20 μl with RNase free water. qRT-PCR was run on a ViiA7 Real-time PCR system, using recommended cycling conditions. Reverse transcription and set-up of qPCR reactions was performed with a QIAgility automated liquid handling system (Qiagen) to decrease variation due to manual pipetting error.

### Statistical analysis

qRT-PCR data was analysed using GenEx qPCR analysis software (Exiqon, Ver 4.4.2.308), taking PCR efficiency into account. GeNorm and Normfinder were used to select appropriate reference genes for normalisation. One-way ANOVA was used to compare the different extraction methods for each tissue and Student’s t-tests (2 tailed, unpaired) used to detect differences in normalised gene expression with *p*-value < 0.05 considered statistically significant.

## Results

### Comparison of yield, purity and integrity of RNA extracted from tissues

For miRNA expression analyses the quality of the RNA is critical. In addition, if there is a limited amount of biological material from which to extract the RNA, researchers may be interested in the yield produced by different RNA extraction methods. Comparison of the yield, quality and integrity of the RNA samples revealed discrete differences with different extraction methods (Table 1). With respect to yield, quantitation of the RNA using a Qubit Fluorometer revealed that the Bioline Isolate II kit consistently recovered lower amounts of RNA, particularly for the brain and liver samples, and the miRNeasy kit consistently gave a high yield of RNA across all samples. For quality, the 260/280 ratios for all samples were acceptable, however the 260/230 ratios were notably lower for the Isolate II kit, indicating possible guanidinium thiocyanate carryover. The average RNA integrity (RIN) was acceptable for both the brain and liver tissues for all tissues, regardless of the kit used. For the lung samples, the RNA integrity was low across all kits, particularly in the Norgen total RNA samples. Multiple peaks were detected in the Bioanalyzer analysis including an additional peak after 60 s (Additional file 3: Figure S4). This is also evident to some degree in the Norgen liver and miRVana lung samples, albeit at a much lesser extent. The RINs for TRIzol lung samples were also very low, with multiple peaks detected. For miRNA enrichment, the miRVana and Norgen total RNA kits consistently yielded higher percentages of miRNA than other methods.

**Table 1:** Analysis of RNA extracted from mouse tissues

Sample	Method	RNA (ng/μl)	260/280	260/230	RIN	miRNA (%)
Brain	Isolate II	26.1 (9.5)	2.20 (0.01)	1.28 (0.34)	8.77 (0.21)	5.94 (1.31)
Brain	miRVana	211.2 (126.2)	2.02 (0.03)	1.54 (0.61)	8.93 (0.59)	24.35 (3.60)
Brain	Norgen total	140.1 (83.7)	2.03 (0.06)	1.86 (0.43)	9.47 (0.38)	17.24 (8.58)
Brain	miRNeasy	280 (32.0)	1.98 (0.01)	1.94 (0.12)	9.07 (0.31)	9.45 (1.82)
Brain	TRIzol	178.3 (116.4)	1.77 (0.06)	2.40 (0.02)	8.07 (0.51)	9.73 (3.49)
Liver	Isolate II	276.7 (167)	2.06 (0.01)	1.47 (0.58)	7.65 (0.49)	12.17 (1.14)
Liver	miRVana	720 (244.9)	2.04 (0.03)	1.88 (0.06)	8.03 (0.94)	17.55 (1.22)
Liver	Norgen total	904 (306.4)	1.99 (0.03)	1.62 (0.18)	8.10 (2.12)	16.83 (2.22)
Liver	miRNeasy	1744 (211.7)	2.01 (0.02)	1.87 (0.25)	7.50 (0.66)	11.06 (3.32)
Liver	TRIzol	1824 (483.2)	1.95 (0.01)	2.04 (0.04)	7.23 (0.49)	5.27 (0.47)
Lung	Isolate II	132.9 (79.8)	2.06 (0.03)	1.28 (0.39)	7.17 (0.31)	5.39 (1.55)
Lung	miRVana	177.3 (22.3)	1.97 (0.02)	1.56 (0.50)	7.95 (1.34)	27.39 (6.16)
Lung	Norgen total	128.7 (12.9)	1.96 (0.01)	2.11 (0.09)	N.D.	37.48 (2.17)
Lung	miRNeasy	262.7 (53.1)	1.97 (0.01)	2.01 (0.01)	6.80 (0.89)	15.42 (0.77)
Lung	TRIzol	101.6 (20.4)	1.70 (0.03)	2.49 (0.03)	5.37 (0.67)	14.98 (1.72)

Average measurements from *n* = 3 mice, with standard deviations in brackets

### microRNA expression in mouse tissues extracted with different kits

**Fig. 3:**
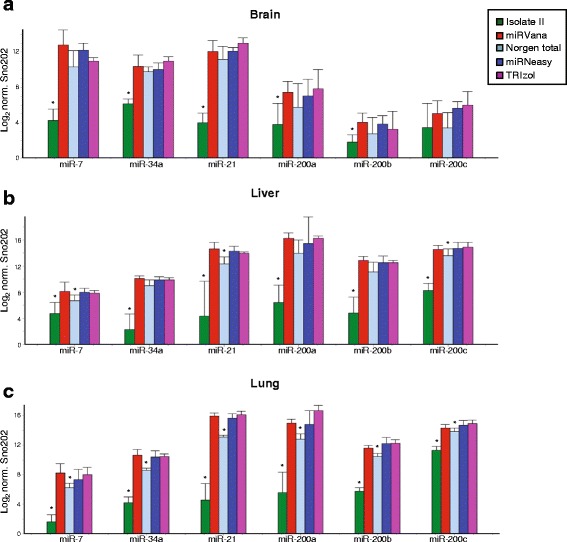
Quantitative real-time PCR analyses of 6 miRNAs comparing different RNA extraction methods. RNA was extracted from murine brain (**a**), liver (**b**) and lung (**c**) tissues using the five indicated methods and endogenous miRNA expression quantitated using TaqMan MicroRNA Assays via qRT-PCR. miRNA expression was normalised using sno202 as a reference gene. Values indicate the average normalised miRNA expression (technical duplicates from triplicate biological isolations) in different tissues on a Log2 scale with SEM error bars. * *p* < 0.05 when compared to three or more other methods

miRNA expression can be analysed by a number of methods including qRT-PCR, microarray hybridization platforms (oligonucleotide array, gene CHIP, Exiqon miRCURY), high throughput sequencing using libraries for small RNAs and nanostring technology based on the nCounter analysis system. However, the gold-standard for analysing miRNA expression is qRT-PCR using specific primers such as Taqman miRNA primers (Thermo Fisher Scientific) (most common method used), locked nucleic acid primers (Exiqon) and poly(A) tailing (Qiagen, Stratagene). In this study, miRNA expression was analysed using Taqman miRNA assays which employ a sequence specific stem-loop primer. We chose to look at six miRNAs (see Additional file 5: Figure S1) that differed in GC content (35–50%), secondary structure (ΔG = − 3.6 – 0.5) and were especially applicable to cancer studies given their tumour suppressive (miR-7-5p, miR-34a-5p) or oncogenic (miR-21-5p) functions, or their involvement in epithelial-mesenchymal transition (EMT, miR-200 family) [17–23]. Furthermore, the inclusion of the three miRNAs from the miR-200 family were also of interest as previous studies have shown that as few as 1–2 nucleotide base changes can drastically effect miRNA profiling results [24]. We found that the Isolate II RNA samples had consistently lower miRNA expression for each of the six miRNAs, in each of the tissues analysed (Fig. 3). Norgen total RNA samples also had significantly lower expression of most miRNAs in liver and lung preparations (Fig. 3b and c). miRNA expression was very comparable across miRVana, miRNeasy and TRIzol extraction methods, regardless of the tissue being analysed.

**Fig. 4:**
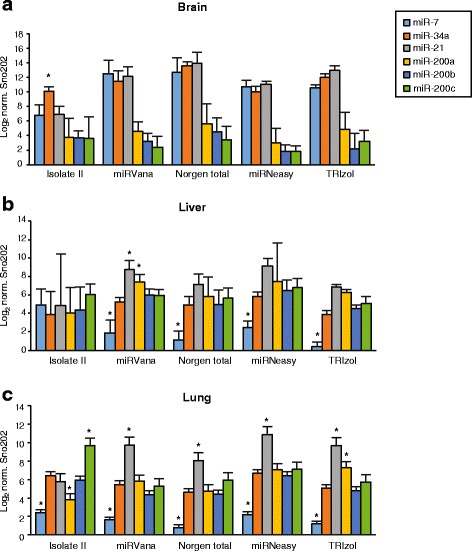
Quantitative real-time PCR analyses comparing miRNA expression patterns in tissues using different extraction methods. Fold abundance of miRNAs quantitated using TaqMan MicroRNA Assays via qRT-PCR from brain (**a**), liver (**b**) and lung (**c**) tissues. miRNA expression was normalised using sno202 as a reference gene. Values indicate the average normalised miRNA expression in different tissues on a Log2 scale with SEM error bars. * *p* < 0.05 when compared to five the other miRNAs for each method

We then took the same data and compared miRNA expression in tissues and stratified by the method used to the extract RNA. This data (Fig. 4) revealed that with the exception of the Isolate II extraction samples, each method exhibited the same trends for the expression of each miRNAs, across the 3 tissues. Using the Isolate II method as compared to the other methods the following was noted: there was less miR-7 and miR-21 recovered from the brain samples; no difference between miRNAs detected in liver; and a bias towards miR-200c recovery compared to miR-21 in lung. Furthermore, there was no significant sequence-specific trend indicating that any bias for low miRNA detection was not sequence related. Loss of recovery of miRNAs with low GC content or highly stable secondary structure using TRIzol to extract RNA has been reported [24]. When we compared our TRIzol data to the other kits (with the exclusion of the Isolate II kit), the data suggests there was less miR-7 and miR-34a compared to miR-21 in brain and liver, and compared to miR-200a in lung. This would lead one to make conclusions that they are significantly different, whereas other methods would not. It is not clear whether GC content or secondary structure is more predictive for differences in recovery.

### miRNA (miR-7 and miR-34a) target gene (EGFR and AXL) expression in tissues

**Fig. 5:**
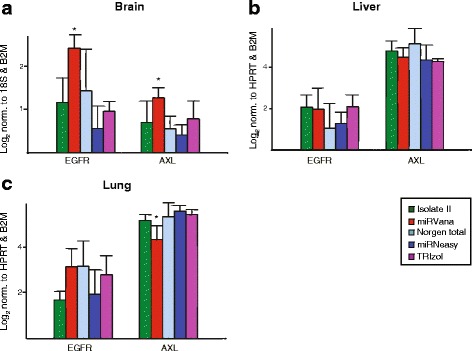
Quantitative real-time PCR analyses of target gene expression from murine tissues. EGFR and AXL expression was analysed from the same RNA samples isolated from brain (**a**), liver (**b**) and lung (**c**) tissues for miRNA detection. Target gene expression was normalised to either: B2M and 18S/HPRT as reference genes. Values indicate the average normalised gene expression (technical duplicates from triplicate isolations) in different tissues on a Log2 scale with SEM error bars. * *p* < 0.05 when compared to three or more other methods

Identification and validation of miRNA target genes is vital to understanding a miRNA’s functional role in normal and disease conditions. Evaluation of miRNA/target gene interactions can be validated via several means: direct validation in in vitro studies with 3′-UTR reporters; through identification of inverse correlations between a miRNA and target gene expression in tissues; or through target gene validation in tissues following miRNA delivery in vivo. Given the latter two scenarios, researchers may be interested in analysing both miRNA and target gene expression in the same RNA preparation. Therefore, we used the same RNA from the 5 extraction methods and evaluated the expression of validated targets of two of the miRNAs, namely EGFR and AXL, targets of miR-7 and miR-34a respectively (Fig. 5). Overall, EGFR expression was much more variable across extraction methods and had more intra-group variation than AXL. For the total RNA kits (Isolate II, Norgen total and TRIzol), expression was comparable for both targets in all three tissues, with the exception of EGFR in the lung with Isolate II. The miRVana kit gave consistently good results with the highest expression of both EGFR and AXL in brain samples, and had high or comparable expression of EGFR and AXL in liver and lung. With the miRNeasy kit EGFR expression in all tissues, and AXL in brain, was notably lower.

**Fig. 6:**
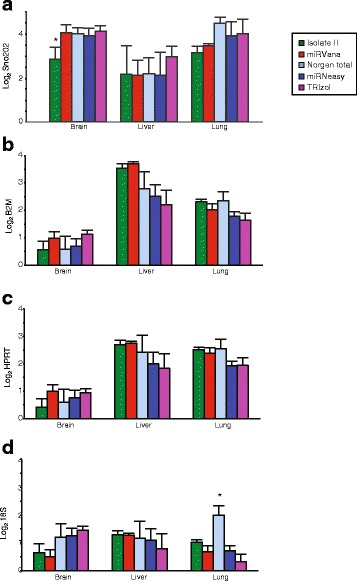
Quantitative real-time PCR analyses of relative house-keeping gene expression in the various murine tissues. Sno202 (**a**), B2M (**b**), HPRT (**c**) and 18S (**d**) expression was assessed via RT-qPCR and GeNorm and Normfinder used to determine suitability for normalisation. Values indicated the average gene expression (technical duplicates from triplicate isolations) with SEM error bars.* *p* < 0.05 when compared to three or more other methods

An important consideration for qPCR is the reference gene used for normalisation. A review of the literature found that snoRNA-202 (sno202) and snoRNA-234 are commonly used for normalisation of mouse tissue samples [25–27]. Furthermore, a profiling study performed by Applied Biosystems analysed expression of potential house-keeping genes in several different mouse tissues including lung, liver and brain and concluded that sno202 produced the least amount of inter and intra-group variation [28]. Therefore, in this study sno202 was used to normalise gene expression and was validated across the extraction methods and different tissues (Fig. 6a). We found sno202 expression was consistent, with the exception of a significant decrease in sno202 expression in brain samples extracted using the Isolate II kit compared to all other kits. Therefore, normalisation of miRNAs to sno202 expression in brain samples extracted with this kit may be further overestimating the abundance of these miRNAs in the original samples. Reference gene expression was also compared for normalisation of target genes (Fig. 6b-d). GeNorm and Normfinder identified 18S and B2M as the best reference genes for brain, whilst HPRT and B2M were best for liver and lung gene expression normalisation. Only 18S expression in lung samples was significantly different to all other RNA samples. As 18S was not used for normalisation it does not account for differences in target gene expression. Furthermore, using multiple reference genes for normalisation minimises technical variation.

## Discussion

In this study we hypothesised that different methods of RNA isolation will impact results when analysing gene expression in tissues. RNA extraction methods can be broadly characterised into either phenol:chloroform extraction followed by alcohol precipitation (TRIzol), phenol:chloroform followed by solid-phase extraction (column-based; miRVana and miRNeasy) and solid-phase separation with/without affinity resin (Norgen total and Isolate II). These methodologies were primarily developed for the extraction of long mRNAs and have been based on the assumption that all RNAs are purified equally. Moreover, there are a number of considerations for choosing a particular RNA extraction method such as the quality, quantity, price, and ease of use (time to extraction). Herein, we present data that shows the method used to extract the RNA will also produce variable results compared to different methods in downstream applications and therefore is another important consideration.

This study demonstrates that RNA isolation methods vary in terms of the quantity and quality of RNA samples, and in the analysis of miRNA and target gene expression. We chose organs that can be difficult to isolate RNA from for different reasons. For example, RNA isolation from brain is often noted to result in low yields due to its high lipid content. This was particularly evident with the Bioline Isolate II kit. The manufacturers’ protocol suggests that up to 5 μg of RNA should be purified from 10 mg of rat/mouse brain. However, the miRNeasy handbook suggests that 5–20 μg of RNA should be achievable from brain with the same amount of input material. Troubleshooting on the manufacturer’s website describes one problem with lipid rich tissues is clogging of the column and suggests decreasing the sample amount or increasing the lysis buffer volume. Our extractions were performed within the suggested limits of the protocol. Subsequently, they suggest performing pre-extraction using TRIsure reagent (Bioline’s equivalent of TRIzol) then column-based separation with the kit to clean-up the aqueous phase. This method would then be comparable to miRVana and miRNeasy RNA extraction methods and may result in better yields for this tissue. Additionally, the RNA quality correlates to tissue-specific responses to physiological stress both prior to and following tissue death. Tissues such as lung and liver characteristically have low RINs and a small 28S peak as these tissues are prone to faster degradation of RNA by high levels of nucleases. To inactivate nucleases the tissue is rapidly frozen and thawing prevented until the weighed material is added to the extraction buffer. Although, thawing of tissue was prevented, we cannot rule out the time from euthanasia of the animal and excision of the organs to rapid-freezing as a contributing factor to the overall low RIN values and some degradation products observed with all lung samples, irrespective of isolation kit used. An alternative method to inactivate nucleases is using a RNA stabilising solution such as RNA*later* (ThermoFisher Scientific), which allows unfrozen tissue samples to be processed later. It may help ensure the integrity of the RNA, but it is not compatible with all RNA isolation procedures and users should check their specific method prior to performing extractions. The addition of a peak after 60 s in some samples in the Bioanalyser analysis suggests gDNA contamination. An additional DNase treatment can be performed on columns with some kits, however a gDNA elimination step is highly recommended during the reverse transcription step. The inclusion of this step in our methods may explain why there was no interference when analysing mRNA expression of the Norgen lung samples, but it did impact miRNA expression analysis as the Taqman miRNA assay protocol does not include gDNA removal. Furthermore, the purity of RNA samples is a critical factor as even though samples with RIN > 7 should perform okay in most downstream applications, those that involve enzymatic reactions such as qPCR can be inhibited by nucleases, metal ions or organic contaminants. In general, the Bioline Isolate II RNA samples had lower 260/230 ratios and contaminants could be contributing to its poor performance. Finally, differences were observed in the enrichment for miRNAs in the total RNA preparation. As miRNAs represent a small fraction of the overall RNA repertoire, it can be difficult to determine their contribution from the integrity analysis. The Qubit microRNA assay offers highly selective detection of low amounts of small RNAs even in the presence of common contaminants. High percentages of miRNAs were commonly detected with miRVana and Norgen extraction methods, however given the integrity analysis for Norgen total RNA preparations, it is possible that the miRNA enrichment may be overestimated as smaller RNA fragments are being detected following degradation or oxidation of larger RNAs (mRNAs, rRNA or tRNAs) or in the case of the lung samples DNA contamination perturbing the Qubit results. Hence, the quantity, quality and purity of RNA samples are highly important factors for choosing a particular RNA extraction method and further research for how to optimise these methods for your tissue of interest can help improve these.

Profiling of miRNAs can give insight as biomarkers for diagnostic or prognostic applications. For example, panels of miRNAs can be used to classify different cancer phenotypes, predict recurrence or response to therapies [1, 12, 29–31]. However, for discovery and clinical application of miRNA-based biomarkers there needs to be optimised and standardised practices for how the RNA is extracted to prevent conflicting results. For example a meta-analysis of 63 published studies by Zhou et al. (2014) found inconsistent and even contrasting results when assessing the prognostic value of the oncomiR, miR-21 [32]. The authors attribute the heterogeneity to differences in the source of the samples, the detection methods and normalization methods but did not allude to the methods for extracting RNA, which we show also contributes.

The biological functions and targets of very few miRNAs have been experimentally validated, but this is critical to realising the potential of miRNA-based therapy. Furthermore, uncovering tissue-specific biological functions will help identify their therapeutic action and potential off-target effects in normal tissues. Validation of miRNA-mRNA interactions is primarily performed in cell culture assays, which involve artificial manipulation of endogenous miRNAs. However, the levels achieved through cell culture manipulation (e.g. transfection of mimics) are not usually at physiological levels observed in vivo therefore, it is important to recapitulate the results in appropriate animal models*.* Currently, no reports have directly compared miRNA and target gene detection from total RNA samples using different extraction methods. We show that RNA extraction methods differ in their efficiency in isolating both short and long RNAs and therefore careful consideration must go into choosing an appropriate method if you wish to detect both from the same sample.

Previous research has commonly assumed that all kinds of RNA are purified equally. However, several reports have emerged indicating differences in extraction efficiency depending on the method used for RNA isolation. Kim et al. (2011) retracted their paper in Molecular Cell as they found that their conclusions about differences in miRNA expression from cells grown at different confluencies (high density versus low density) and when they were detached from the culture dish (adherent versus suspension) were actually accounted for by differences in RNA extraction efficiency using the TRIzol method [24]. They found that miRNAs with low GC content and stable secondary structure were lost during extraction, rather than being degraded in the cells as they had initially published [33]. Previous results from El-Khoury et al. (2016) found differences in miRNA recovery from cells, plasma and urine/plasma-derived exosomes when comparing TRIzol LS, miRNeasy serum/plasma and miRCURY biofluid extraction kits [13]. The authors found that the miRCURY kit isolated highly pure RNA but poorly recovered miRNAs, TRIzol yielded low purity RNA which impacted PCR efficiency, whilst miRNeasy yielded poor quality RNA but performed the best for miRNA detection. Likewise, McAlexander et al. (2013) found differences in miRNA extraction from plasma and cerebrospinal fluid [15]. They compared miRVana, miRCURY Cell and Plant kit and TRIzol LS extractions with and without glycogen as a carrier. miRVana was similar with or without glycogen, while miRCURY without glycogen had slightly lower miRNA recovery than miRVana. However, glycogen greatly improved miRNA recovery with the miRCURY kit but exacerbated the low yield and variability with TRIzol extraction. They then went on to compare miRCURY Cell and Plant kit to miRCURY Biofluids and miRNeasy serum/plasma extraction methods with glycogen as a carrier and found that miRCURY Biofluids had the highest relative abundance of spiked-in exogenous miRNA and concluded this was the superior method for their particular application. Collectively, these studies highlight the differences in RNA recovery using different extraction methods and suggest the need to optimise for your cell, tissue, and/or fluid of interest.

There are several steps during the work-flow that can introduce experimental variation. Beginning with the method for fixing or freezing the tissue, storage of the material, RNA extraction method, method used for reverse transcription and qPCR amplification or other downstream platforms. In this study we have attempted to control for some of these variables by flash-freezing the tissues, storage at − 80 °C, preventing thawing before extraction and using the most recommended practices for miRNA expression analysis. For instance, the use of sequence specific RT primers as opposed to a universal RT primer has been shown to be superior for specific product amplification [7]. qPCR is considered the gold standard for analysing miRNA and target expression due to its sensitivity and specificity over global profiling platforms. Importantly, the quality of the results obtained is more important than the sheer number of miRNAs profiled from large sequencing based platforms. Furthermore, although we did not compare the effect of miRNA enrichment from our RNA samples previous reports have advised against it, particularly for global miRNA profiling platforms. For instance, Redshaw et al. (2013) found that the short RNA enrichment procedure results in significantly less relative miRNA levels when comparing total RNA and enriched material, specifically the enrichment procedure reduced the copy number of miRNAs by up to 25% of that present from pre-enriched samples and that this loss varied for different miRNA sequences. Therefore, miRNA data from total and short RNA preparations may not be directly comparable. Furthermore, it has been suggested that larger RNA can act as a carrier for small RNAs [24]. Whether miRNA enriched methods for all companies would result in better recovery from tissues remains to be determined. Although Bioline suggest a separate kit for miRNA isolation, we used the Isolate II total RNA kit to directly compare with other extraction methods as the miRNA specific kit does not allow isolation of both short and long RNAs from the same elution. Rather the miRNA species are enriched for and isolated first, and then long RNAs can be extracted after, resulting in two separate fractions. Although the Isolate II is not optimised to isolate small RNAs such as the miRVana or miRNeasy kits, it also wasn’t superior for the target gene expression. Given the large discrepancy between certain kits researchers should opt for a more robust extraction method.

## Conclusion

Comparative analysis of five commonly used RNA extraction methods revealed the miRVana kit was the best performer for producing consistently high yield and quality RNA without compromising miRNA and target gene detection levels. Although this kit was the most expensive, its high quality output and ease of use makes it a preferable method for RNA extraction, particulary if researchers wish to use the same RNA preparation for miRNA and gene expression analyses, as the other methods had lower detection levels and more variability. Empirically, this needs to be optimised for a specific application. A final caution to researchers is that just because a kit is described as isolating total RNA and is appropriate for mammalian tissues, cultured cells or biological fluids; it does not mean that it yields all types of RNAs equally.

## Additional files

Additional file 1: **Figure S2.** RNA integrity analysis of samples isolated from brain tissues. RNA was isolated using five different methods and integrity analysed using an Agilent Bioanalyser with Bioanalyser RNA 6000 Nano Assay. RIN, RNA integrity number. (EPS 4419 kb)

Additional file 2: **Figure S3.** RNA integrity analysis of samples isolated from liver tissues. RNA was isolated using five different methods and integrity analysed using an Agilent Bioanalyser with Bioanalyser RNA 6000 Nano Assay. RIN, RNA integrity number. (EPS 3085 kb)

Additional file 3: **Figure S4.** RNA integrity analysis of samples isolated from lung tissues. RNA was isolated using five different methods and integrity analysed using an Agilent Bioanalyser with Bioanalyser RNA 6000 Nano Assay. RIN, RNA integrity number. (EPS 3137 kb)

Additional file 4: **Table S1.** Primers used for qRT-PCR. (DOCX 32 kb)

Additional file 5: **Figure S1.** GC content and predicted secondary structure of miRNAs used in this study. GC content was calculated based on the mature sequence provided in miRBase.org. Secondary structure and thermodynamic stability was determined using Mfold web server for nucleic acid folding and hybridization prediction. (EPS 794 kb)
