# Supplementary material for: Total RNA extraction from tissues for microRNA and target gene expression analysis: not all kits are created equal
Source: BMC Biotechnol. 2018 Mar 16;18:16. doi: 10.1186/s12896-018-0421-6 (PMC5857145; doi:10.1186/s12896-018-0421-6)
Supplement: Supplementary file 4 — Table S1. Primers used for qRT-PCR. (DOCX 32 kb) [file 12896_2018_421_MOESM4_ESM.docx]

**Supplementary Table 1: Primers used for qRT-PCR**

| Primer assay name | Company | Catalogue number |
| --- | --- | --- |
| dme-miR-7 | Thermo Fisher Scientific | 4427975, 000268 |
| hsa-miR-34a | Thermo Fisher Scientific | 4427975, 000426 |
| hsa-miR-21 | Thermo Fisher Scientific | 4427975, 000397 |
| hsa-miR-200a | Thermo Fisher Scientific | 4427975, 000502 |
| hsa-miR-200b | Thermo Fisher Scientific | 4427975, 002251 |
| hsa-miR-200c | Thermo Fisher Scientific | 4427975, 002300 |
| snoRNA202 | Thermo Fisher Scientific | 4427975, 001232 |
| Mm_Egfr_1_SG | Qiagen | 249900, QT00101584 |
| Mm_Axl_1_SG | Qiagen | 249900, QT00101353 |
| Mm_B2m_2_SG | Qiagen | 249900, QT01149547 |
| Mm_Hprt_1_SG | Qiagen | 249900, QT00166768 |
| Mm_Rn18s_3_SG | Qiagen | 249900, QT02448075 |
